# Functionalized silicon quantum dots by *N*-vinylcarbazole: synthesis and spectroscopic properties

**DOI:** 10.1186/1556-276X-9-384

**Published:** 2014-08-07

**Authors:** Jianwei Ji, Guan Wang, Xiaozeng You, Xiangxing Xu

**Affiliations:** 1 State Key Laboratory of Coordination Chemistry, Nanjing National Laboratory of Microstructures, School of Chemistry and Chemical Engineering, Nanjing University, Nanjing 210093, People's Republic of China

**Keywords:** Silicon quantum dots, *N*-vinylcarbazole, Surface modification, Spectroscopic property

## Abstract

Silicon quantum dots (Si QDs) attract increasing interest nowadays due to their excellent optical and electronic properties. However, only a few optoelectronic organic molecules were reported as ligands of colloidal Si QDs. In this report, *N*-vinylcarbazole - a material widely used in the optoelectronics industry - was used for the modification of Si QDs as ligands. This hybrid nanomaterial exhibits different spectroscopic properties from either free ligands or Si QDs alone. Possible mechanisms were discussed. This type of new functional Si QDs may find application potentials in bioimaging, photovoltaic, or optoelectronic devices.

## Background

Silicon (Si) is one of the most important semiconductor materials for the electronics industry. The energy structure of bulk Si is indirect bandgap, which is greatly changed by the quantum confinement effect for small enough Si nanocrystals (NCs) called Si quantum dots (QDs), making Si QDs fluorescent with a tunable spectrum. Excellent spectroscopic properties, such as high quantum yield, broad absorption window, and narrow fluorescent wavelength, contribute to a rapid development in Si QD research [1]. Nontoxicity to the environment and the use of an economic source material are other two merits for the application of Si QDs in optoelectronics [2,3], solar energy conversion [4,5], biology [6-8], splitting water [9], etc. Si QDs can be prepared using a variety of techniques such as wet chemical reduction [10-18], metathesis reaction [19], disproportionation reaction [20,21], thermal annealing of Si-rich SiC [22], electrochemical etching [23], plasma synthesis or plasma-enhanced chemical vapor deposition (PECVD) [24-27], and high-temperature hydrogen reduction method [28-32]. Because Si QDs are chemically active, their surface should be passivated for further use. Molecules with alkyl chains and -CH_3_, -COOH, or -NH_2_ ends have been widely employed as surface ligands to enhance the stability of Si QDs [28-36]. These ligands help prevent the oxidation of silicon and enhance the dispersibility of Si QDs in organic or aqueous solution. In addition to the surface protection, optoelectronic functional molecules as ligands of Si QDs are attracting increasing interest in recent years for the crucial role of the ligands to the interfacial related process in optoelectronic or light-harvesting devices. Kryschi and co-workers showed that 3-vinylthiophene ligands may act as surface-bound antennae that mediate ultrafast electron transfer or excitation energy transfer across the Si QD interface via high-energy two-photon excitation [37,38]. They also reported that for 2- and 4-vinylpyridine-terminated Si QDs, ultrafast excitation relaxation dynamics involving decay and rise dynamics faster than 1 ps were ascribed to electronic excitation energy transfer from an initially photoexcited ligand state to Si QD conduction band states [39]. Larsen and Kauzlarich and their co-workers investigated the transient dynamics of 3-aminopropenyl-terminated Si QDs [40]. A formation and decay of a charge transfer excited state between the delocalized π electrons of the carbon linker and the Si core excitons were proposed to interpret one-photon excitation. Zuilhof et al. reported Si QDs functionalized with a red-emitting ruthenium complex to exhibit Förster resonance energy transfer (FRET) from Si QDs to the complex [41]. The ligands on the Si surface may also induce optoelectronic interactions to other QDs such as CdSe QDs, e.g., Sudeep and Emrick found that hydrosilylation of Si QDs provides a corona of phosphine oxides that may serve as ligands for CdSe QDs [42]. This surface functionalization of the Si QDs was proved a key to the photoluminescence quenching of CdSe QDs, as conventional (alkane-covered) Si QD samples give no evidence of such optoelectronic interactions. Recently, we reported 9-ethylanthracene-modified Si QDs showing dual emission peaks that originate from the Si QD core and the ligands [43]. In this report, we demonstrate the synthesis and surface modification of Si QDs with *N*-ethylcarbazole, using hydrogen-terminated Si QDs and *N*-vinylcarbazole as the starting materials. Both anthracene and carbazole are fluorescent molecules and organic semiconductors. The main difference is that anthracene is an electron transport material while carbazole is a hole transport material. This difference is important for the structure design of optoelectronic or photovoltaic devices utilizing these Si QD-based hybrid materials. *N*-vinylcarbazole and its derivatives as a class of typical optoelectronic molecules show abundant attractive properties and can be applied in dye, optics, electronics, and biology [44-48]. *N*-vinylcarbazole is also the monomer precursor of poly(*N*-vinylcarbazole) (PVK) polymer which is widely used as a hole transport or electroluminescent material in organic optoelectronic devices [49-51]. The *N*-ethylcarbazole-modified Si QDs (referred to as ‘N-ec-Si QDs’ for short) exhibit photoluminescence quite different from freestanding *N*-vinylcarbazole- or hydrogen-modified Si QDs. This hybrid nanomaterial was characterized and investigated by powder X-ray diffraction (XRD), transmission electron microscopy (TEM), Fourier transform infrared spectroscopy (FTIR), photoluminescence (PL), and PL lifetime measurement.

## Methods

### Materials and equipment

*N*-vinylcarbazole (98%), HSiCl_3_ (99%), and mesitylene (97%) were purchased from Aladdin Reagent Co., Ltd. (Shanghai, China). Analytical-grade ethanol (99.5%) and hydrofluoric acid (40% aqueous solution) were received from Sinopharm Chemical Reagent Co., Ltd. (SCRC; Shanghai, China). All reagents were used as purchased without further purification. The XRD spectrum was performed on a Bruker D8 Advance instrument (Bruker AXS GmbH, Karlsruhe, Germany) with Cu Kα radiation (*λ* = 1.5418 Å). TEM images were obtained on a JEM-2100 transmission electron microscope with an acceleration voltage of 200 kV (JEOL, Ltd., Akishima, Tokyo, Japan). The FTIR spectra were measured by a Bruker VECTOR 22 spectrometer (Bruker, Germany) with KBr pellets. The PL and excitation spectra were collected by a Hitachi F-4600 fluorescence spectrophotometer (Hitachi, Ltd., Chiyoda-ku, Japan). The UV-vis absorption spectra were measured by a Shimadzu UV-2700 UV-vis spectrophotometer (Shimadzu Corporation, Kyoto, Japan). The PL lifetime was obtained on a Zolix Omni-λ 300 fluorescence spectrophotometer (Zolix Instruments Co., Ltd., Beijing, China).

### Synthesis of hydrogen-terminated Si QDs

Si QDs were synthesized by reduction of (HSiO_1.5_)_*n*_ powder with hydrogen [28,29]. Typically, 5 mL of HSiCl_3_ (49.5 mmol) was added to a three-neck flask equipped with a mechanical stir bar, cooled to −78°C in an ethanol bath, and kept for 10 min, using standard Schlenk techniques with N_2_ protection. With the injection of 20 mL H_2_O by a syringe, a white precipitate formed immediately. After 10 min, the white (HSiO_1.5_)_*n*_ was collected by centrifugation, washed by distilled water, and dried in vacuum at 60°C. In the reduction step, (HSiO_1.5_)_*n*_ (1.10 g) was placed in a corundum crucible and transferred to a tube furnace. The sample was heated to 1,150°C and maintained for 1.5 h with a heating rate of 5°C/min under a slightly reducing atmosphere containing 5% H_2_ and 95% Ar (≥99.999%). After cooling to room temperature, a light brown product of Si/SiO_2_ composite was collected. The Si/SiO_2_ composite (50 mg) was grinded with a mortar and pestle for 10 min. Then the powder was transferred to a Teflon container (20 mL) with a magnetic stir bar. A mixture of ethanol (1.5 mL) and hydrofluoric acid (40%, 2.5 mL) was added. The light brown mixture was stirred for 60 min to dissolve the SiO_2_. Finally, 5 mL mesitylene was added to extract the hydrogen-terminated Si QDs into the upper organic phase, forming a brown suspension (A), which was isolated for further surface modification.

### Modification of Si QDs by functional organic molecules

*N*-vinylcarbazole (1 mmol) was dissolved in 15 mL mesitylene and loaded in a 50-mL three-neck flask equipped with a reflux condenser. Then 2 mL Si QDs (A) was injected by a syringe. The mixture was degassed by a vacuum pump for 10 min to remove any dissolved gases from the solution. Protected by N_2_, the solution was heated to 156°C and kept for 12 h. After cooling to room temperature, the resulting Si QDs were purified by vacuum distillation and then washed by ethanol to remove excess solvent and organic ligands. The as-prepared brown solid product was readily re-dispersed in mesitylene to give a yellow solution.

## Results and discussion

**Figure 1:**
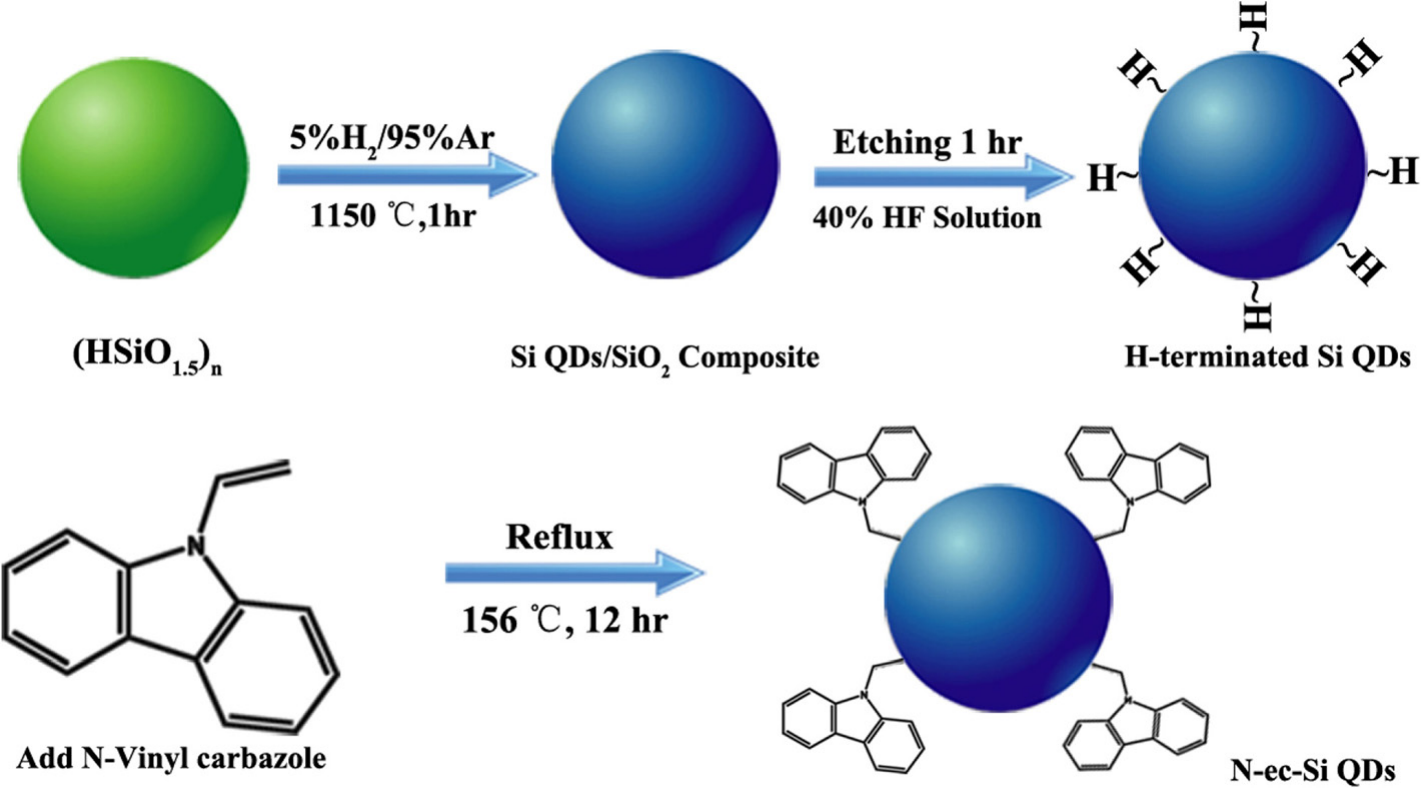
Synthetic strategy of N-ec-Si QDs.

The synthesis route of N-ec-Si QDs is summarized in Figure 1. The HSiCl_3_ hydrolysis product (HSiO_1.5_)_*n*_ was reduced by H_2_ at 1,150°C for 1.5 h. In this step, the temperature and time are crucial in controlling the size of Si QDs. The higher the temperature and the longer the reduction time, the bigger the sizes of Si QDs. The following HF etching procedure also plays a key role for the size tuning of the Si QDs. HF not only eliminates the SiO_2_ component and liberates the free Si QDs but also etches Si QDs gradually. Another contribution of HF etching is the modification of the surface of Si QDs with hydrogen atoms in the form of Si-H bonds, which can be reacted with an ethylenic bond or acetylenic bond to form a Si-C covalent bond [28-32].

**Figure 2:**
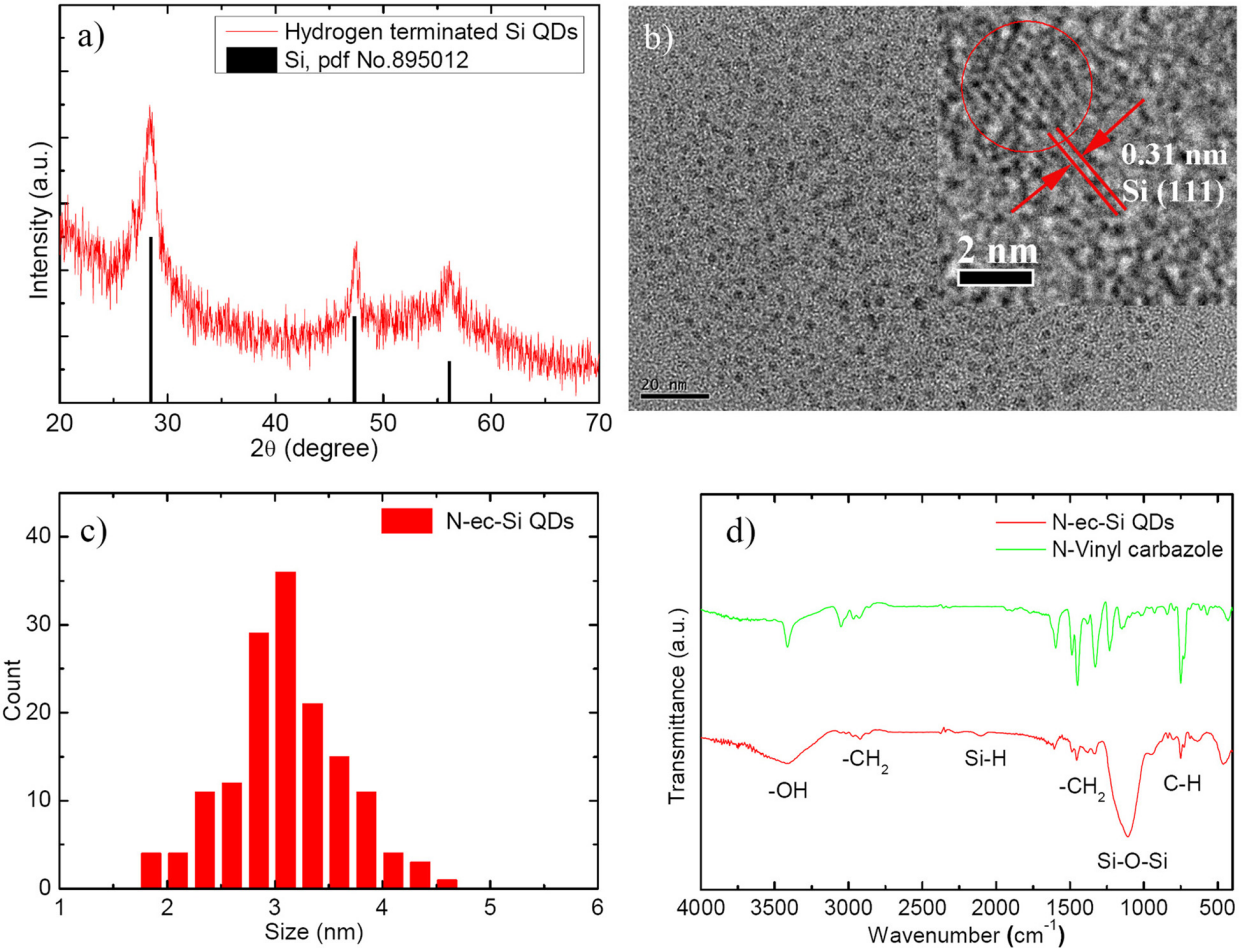
**Characterization of Si QDs and N-ec-Si QDs. (a)** XRD pattern of the hydrogen-terminated Si QDs. **(b)** TEM image and HRTEM image (inset) of the N-ec-Si QDs (scale bar 20 nm, inset 2 nm). **(c)** Size distribution of the N-ec-Si QDs. **(d)** FTIR spectra of the N-ec-Si QDs and pure *N*-vinylcarbazole.

The hydrogen-terminated Si QDs are characterized by XRD (Figure 2a). The XRD pattern shows broad reflections (2*θ*) centered at around 28°, 47°, and 56°, which are readily indexed to the {111}, {220}, and {311} crystal planes, respectively, consistent with the face-centered cubic (fcc)-structured Si crystal (PDF No. 895012). Figure 2b and its inset show typical TEM and high-resolution TEM (HRTEM) images of N-ec-Si QDs, respectively. A *d*-spacing of approximately 0.31 nm is observed for the Si QDs by HRTEM. It is assigned to the {111} plane of the fcc-structured Si. The size distribution of N-ec-Si QDs measured by TEM reveals that the QD sizes range from 1.5 to 4.6 nm and the average diameter is about 3.1 nm (Figure 2c). In the FTIR spectrum of N-ec-Si QDs, a series of characteristic vibrations from Si QDs and carbazole are observed (Figure 2d). The weak vibration resonance centered at 2,090 cm^−1^ can be assigned to the coupled H-Si-Si-H stretching or monohydride Si-H bonds. This result shows that the Si-H bonds were only partially replaced by Si-C because of the rigid and steric effect of the *N*-vinylcarbazole molecule. Compared to the IR spectrum of *N*-vinylcarbazole, similar vibrational peaks can be found in the spectrum of N-ec-Si QDs. The CH_2_ symmetric and asymmetric stretching vibrations in the range 2,920 to 2,850 cm^−1^, the CH_2_ bending vibration at approximately 1,450 cm^−1^, and the aromatic group vibration bands at approximately 750 cm^−1^ can be assigned to the surface-modified *N*-ethylcarbazole (-NC_14_H_12_) ligands. This indicates the successful modification of *N*-vinylcarbazole onto the Si QDs. It should be noticed that the Si-O-Si vibration band at 1,000 to 1,200 cm^−1^ is recorded, suggesting possible oxidation of the Si QD surface. This may due to the steric effect of carbazole, that is, the Si QD surface cannot be fully protected by the ligand, in which some Si-H remained and encountered oxidation when exposed to air.

**Figure 3:**
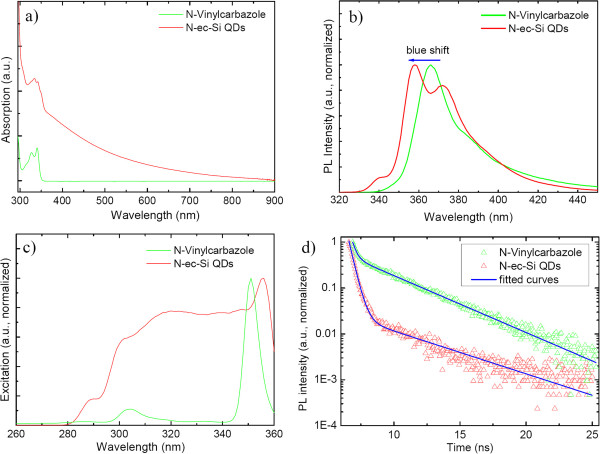
**Spectroscopic properties of N-ec-Si QDs and *N*-vinylcarbazole in mesitylene solution. (a)** UV spectra. **(b)** Photoluminescence spectra. **(c)** Excitation spectra. **(d)** PL decay curves. (excitation at 302 nm; emissions of 358 nm for N-ec-Si QDs and 366 nm for *N*-vinylcarbazole were adopted for the excitation spectra measurement).

Figure 3a shows the absorption spectra of *N*-vinylcarbazole and N-ec-Si QDs. The absorption band at 320 to 360 nm of the N-ec-Si QDs is assigned to the carbazole ligand. It suggests that ligands can be employed to enhance the absorption of pure Si QDs, therefore providing a potential strategy to increase the light-harvesting efficiency of QDs in solar cells [52,53]. Upon excitation at 302 nm, the N-ec-Si QDs and *N*-vinylcarbazole show intense emission bands at approximately 358 nm and approximately 366 nm, respectively (Figure 3b). In comparison with *N*-vinylcarbazole, the emission in the 9-ea-Si QDs exhibits a blueshift of 8 nm and a shoulder peak at approximately 372. When carbazole was linked to the surface of Si QDs by Si-C bond by the hydrosilylation reaction, the vinyl group in *N*-vinylcarbazole was transformed into an ethyl group. Therefore, the conjugate system of the molecule reduced from *N*-vinylcarbazole to carbazole, inducing a bigger electronic bandgap. In addition, the ligand to QD bonding would enhance the structural rigidity of the ligand. These reasons may contribute to the blueshift of the PL spectrum. Commonly, the extension of molecular conjugated orbitals of a ligand to the attached materials would lead to a redshift. In N-ec-Si QDs, the ethyl group formed through the hydrosilylation reaction separates the conjugated part, the carbazole group, from the silicon nanocrystal, which prevents or weakens the interaction of the carbazole group with the electronic wave functions of the Si QDs. Therefore, a redshift is prohibited. A similar blueshift was also demonstrated in our recent work for 9-ethylanthracene modified on Si QDs [43].

The N-ec-Si QDs and *N*-vinylcarbazole show distinct excitation spectra within the range of 280 to 360 nm (Figure 3c), indicating that the energy structure of N-ec-Si QDs is different from *N*-vinylcarbazole. PL decay curves of N-ec-Si QDs and *N*-vinylcarbazole were investigated at room temperature in mesitylene solution (Figure 3d). The PL decay curves are fitted to the exponential function

(1) $$I\left(t\right)=\sum_{i=1}^{n}A_{i}\exp\left(-\left(t-t_{0}\right)/\tau_{i}\right)$$

where *τ*_*i*_ is the PL decay lifetime, *A*_i_ is the weighting parameter, and *n* = 2. The fitting parameters are given in Table 1. The average lifetime is determined by the equation [54]

**Table 1:** Fitting parameters of the PL decay curves

**Sample**	**Emission (nm)**	***τ***_**1 **_**(ns)**	***τ***_**2 **_**(ns)**	***a***_**1**_^**a**^	***a***_**2**_^**a**^	***R***^**2**^	***τ***_**av **_**(ns)**
*N*-vinylcarbazole	366	0.27	3.5	0.58	0.42	0.998	3.2
N-ec-Si QDs	358	0.35	4.6	0.98	0.02	0.997	1.4

^a^$a_{i}=\frac{A_{i}}{\sum_{j=1}^{n}A_{j}}$, *i* = 1, 2, *n* = 2.

(2) $$\tau_{\mathrm{av}}\;=\;\sum_{i=1}^{n}(A_{i}\tau_{i}^{2})/\sum_{i=1}^{n}(A_{i}\tau_{i})$$

**Figure 4:**
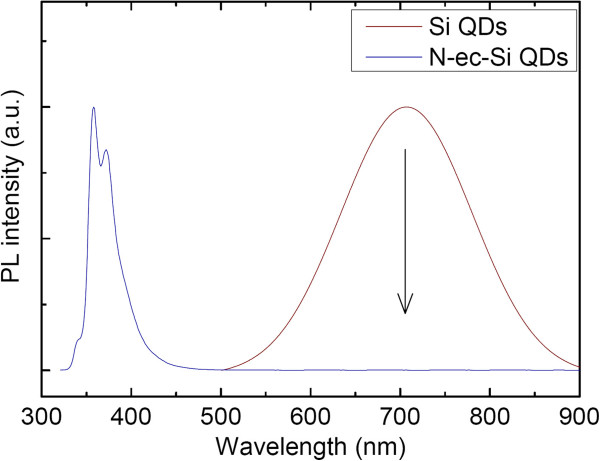
Photoluminescence spectra of N-ec-Si QDs (excitation 302 nm) and hydrogen-modified Si QDs (excitation 360 nm).

The average PL decay lifetime of N-ec-Si QDs is 1.4 ns, much shorter than that of *N*-vinylcarbazole which is 3.2 ns. The lifetime diversity may be influenced by many factors. First, the hydrosilylation reaction induces the transformation of the molecule structure. Second, the *N*-vinylcarbazole dispersion state in the mesitylene is not clear. Possible π-π packing of the molecules may lead to a redshift. Support can be found in the fact that N-ec-Si QDs show a more symmetric PL spectrum to the absorption spectrum than *N*-vinylcarbazole exhibits. Third, the interaction of the ligands with the Si-QDs and interaction between the modified ligands are inevitably encountered [55]. Additionally, the oxidation of the silicon surface may induce additional non-radiative passways for the excitation. All of these factors would lead to PL lifetime shortening [56]. Unlike alkyl ligands or 9-ethylanthracene-modified Si QDs, the fluorescence from hydrogen-terminated Si QDs was quenched after the carbazole modification (Figure 4). It may be induced by the interaction of carbazole with the Si QDs. The fluorescence quantum yield of *N*-vinylcarbazole and N-ec-Si QDs was estimated to be 26.6% and 11.2%, respectively, by using Coumarin 540 dye in methanol as a reference (91%) [57]. The decrease of the quantum yield could be a result from fast non-radiative relaxation of the excited states, induced by the interaction of the ligands to Si QDs or surface states, which also could be an interpretation for the lifetime shortening. From the molecular design aspect, the functional group modified by a long alkyl tail with an ethyl or vinyl end would be an ideal ligand structure in which the Si QDs and the functional group are spatially separated. Also, the flexibility of the long alkyl chain exhibits a smaller steric effect. The surface of Si QDs could be more effectively protected, thus preserving the fluorescence of the Si QD core.

## Conclusions

In conclusion, N-ec-Si QDs were successfully prepared and characterized. Spectroscopic properties were investigated and discussed. The absorption, excitation, PL, and PL decay properties of *N*-ethylcarbazole ligands on the Si QD surface are significantly different from those of *N*-vinylcarbazole in solution. Hopefully, the synthesis strategy could be extended for the syntheses of a series of Si QDs containing various optoelectronic functional organic ligands, with application potentials ranging from optic, electronic, and photovoltaic devices to biotechnology.

## Competing interests

The authors declare that they have no competing interests.

## Authors’ contributions

JWJ and GW contributed equally to the manuscript. XZY and XXX designed the research. JWJ, GW, and XXX carried out the experiments and drafted the manuscript. All authors read and approved the final manuscript.

## References

[B1] Veinot JGC Synthesis, surface functionalization, and properties of freestanding silicon nanocrystals Chem Commun 2006 9 4160 10.1039/b607476f 17031422

[B2] Puzzo DP Henderson EJ Helander MG Wang ZB Ozin GA Lu ZH Visible colloidal nanocrystal silicon light-emitting diode Nano Lett 2011 9 1585 21443164 10.1021/nl1044583

[B3] Cheng KY Anthony R Kortshagen UR Holmes RJ High-efficiency silicon nanocrystal light-emitting devices Nano Lett 2011 9 1952 21462935 10.1021/nl2001692

[B4] Yuan GB Aruda K Zhou S Levine A Xie J Wang DW Understanding the origin of the low performance of chemically grown silicon nanowires for solar energy conversion Angew Chem Int Ed 2011 9 2334 10.1002/anie.201006617 21351348

[B5] Liu CY Kortshagen UR A silicon nanocrystal Schottky junction solar cell produced from colloidal silicon nanocrystals Nanoscale Res Lett 2010 9 1253 20676200 10.1007/s11671-010-9632-z PMC2897035

[B6] Pacholski C Sartor M Sailor MJ Cunin F Miskelly GM Biosensing using porous silicon double-layer interferometers: reflective interferometric Fourier transform spectroscopy J Am Chem Soc 2005 9 11636 16104739 10.1021/ja0511671 PMC2568989

[B7] He Y Kang ZH Li QS Tsang CHA Fan CH Lee ST Ultrastable, highly fluorescent, and water-dispersed silicon-based nanospheres as cellular probes Angew Chem Int Ed 2009 9 128 10.1002/anie.200802230 18979474

[B8] Stanca L Petrache SN Serban AI Staicu AC Sima C Munteanu MC Zărnescu O Dinu D Dinischiotu A Interaction of silicon-based quantum dots with gibel carp liver: oxidative and structural modifications Nanoscale Res Lett 2013 9 254 23718202 10.1186/1556-276X-8-254 PMC3680243

[B9] Erogbogbo F Lin T Tucciarone PM LaJoie KM Lai L Patki GD Prasad PN Swihart MT On-demand hydrogen generation using nanosilicon: splitting water without light, heat, or electricity Nano Lett 2013 9 451 23317111 10.1021/nl304680w

[B10] Heath JR A liquid-solution-phase synthesis of crystalline silicon Science 1992 9 1131 17789084 10.1126/science.258.5085.1131

[B11] Bley RA Kauzlarich SM A low-temperature solution phase route for the synthesis of silicon nanoclusters J Am Chem Soc 1996 9 12461

[B12] Dhas NA Raj CP Gedanken A Preparation of luminescent silicon nanoparticles: a novel sonochemical approach Chem Mater 1998 9 3278

[B13] Wilcoxon JP Samara GA Tailorable, visible light emission from silicon nanocrystals App Phys Lett 1999 9 3164

[B14] Baldwin RK Pettigrew KA Ratai E Augustine MP Kauzlarich SM Solution reduction synthesis of surface stabilized silicon nanoparticles Chem Commun 2002 9 1822 10.1039/b205301b 12271626

[B15] Warner JH Hoshino A Yamamoto K Tilley RD Water-soluble photoluminescent silicon quantum dots Angew Chem Int Ed 2005 9 4550 10.1002/anie.200501256 15973756

[B16] Tilley RD Yamamoto K The microemulsion synthesis of hydrophobic and hydrophilic silicon nanocrystals Adv Mater 2006 9 2053

[B17] Rosso-Vasic M Spruijt E van Lagen B Cola LD Zuilhof H Alkyl-functionalized oxide-free silicon nanoparticles: synthesis and optical properties Small 2008 9 1835 18752208 10.1002/smll.200800066

[B18] Lin SW Chen DH Synthesis of water-soluble blue photoluminescent silicon nanocrystals with oxide surface passivation Small 2009 9 72 18985673 10.1002/smll.200800677

[B19] Pettigrew KA Liu Q Power PP Kauzlarich SM Solution synthesis of alkyl- and alkyl/alkoxy-capped silicon nanoparticles via oxidation of Mg_2_Si Chem Mater 2003 9 4005

[B20] Liu SM Sato S Kimura K Synthesis of luminescent silicon nanopowders redispersible to various solvents Langmuir 2005 9 6324 15982038 10.1021/la050346t

[B21] Liu SM Yang Y Sato S Kimura K Enhanced photoluminescence from Si nano-organosols by functionalization with alkenes and their size evolution Chem Mater 2006 9 637

[B22] Wan ZY Huang SJ Green MA Conibeer G Rapid thermal annealing and crystallization mechanisms study of silicon nanocrystal in silicon carbide matrix Nanoscale Res Lett 2011 9 129 21711625 10.1186/1556-276X-6-129 PMC3211175

[B23] Carter RS Harley SI Power PP Augustine MP Use of NMR spectroscopy in the synthesis and characterization of air- and water-stable silicon nanoparticles from porous silicon Chem Mater 2005 9 2932

[B24] Jurbergs D Rogojina E Mangolini L Kortshagen U Silicon nanocrystals with ensemble quantum yields exceeding 60% Appl Phys Lett 2006 9 2331161

[B25] Kortshagen U Mangolini L Bapat A Plasma synthesis of semiconductor nanocrystals for nanoelectronics and luminescence applications J Nanoparticle Res 2007 9 39

[B26] Lin GR Lin CJ Lin CT Low-plasma and high-temperature PECVD grown silicon-rich SiO_*x*_ film with enhanced carrier tunneling and light emission Nanotechnol 2007 9 395202 10.1088/0957-4484/18/39/395202 21730413

[B27] Lin GR Lin CJ Kuo HC Lin HS Kao CC Anomalous microphotoluminescence of high-aspect-ratio Si nanopillars formatted by dry-etching Si substrate with self-aggregated Ni nanodot mask Appl Phys Lett 2007 9 143102

[B28] Henderson EJ Veinot JGC From phenylsiloxane polymer composition to size-controlled silicon carbide nanocrystals J Am Chem Soc 2009 9 809 19140800 10.1021/ja807701y

[B29] Henderson EJ Kelly JA Veinot JGC Influence of HSiO1.5 sol–gel polymer structure and composition on the size and luminescent properties of silicon nanocrystals Chem Mater 2009 9 5426

[B30] Mastronardi ML Hennrich F Henderson EJ Maier-Flaig F Blum C Reichenbach J Lemmer U Kübel C Wang D Kappes MM Ozin GA Preparation of monodisperse silicon nanocrystals using density gradient ultracentrifugation J Am Chem Soc 2011 9 11928 21740050 10.1021/ja204865t

[B31] Mastronardi ML Maier-Flaig F Faulkner D Henderson EJ Kübel C Lemmer U Ozin GA Size-dependent absolute quantum yields for size-separated colloidally-stable silicon nanocrystals Nano Lett 2012 9 337 22195549 10.1021/nl2036194

[B32] Hessel CM Reid D Panthani MG Rasch MR Goodfellow BW Wei J Fujii H Akhavan V Korgel BA Synthesis of ligand-stabilized silicon nanocrystals with size-dependent photoluminescence spanning visible to near-infrared wavelengths Chem Mater 2012 9 393

[B33] Sieval AB Linke R Zuilhof H Sudhölter EJR High-quality alkyl monolayers on silicon surfaces Adv Mat 2000 9 1457

[B34] Buriak JM Organometallic chemistry on silicon and germanium surfaces Chem Rev 2002 9 1271 11996538 10.1021/cr000064s

[B35] Shirahata N Hozumi A Yonezawa T Monolayer-derivative functionalization of non-oxidized silicon surfaces Chem Rec 2005 9 145 15889408 10.1002/tcr.20041

[B36] Boukherroub R Chemical reactivity of hydrogen-terminated crystalline silicon surfaces Curr Op Sol St Mat Sci 2005 9 66

[B37] Cimpean C Groenewegen V Kuntermann V Sommer A Kryschi C Ultrafast exciton relaxation dynamics in silicon quantum dots Laser Photonics Rev 2009 9 138

[B38] Groenewegen V Kuntermann V Haarer D Kunz M Kryschi C Excited-state relaxation dynamics of 3-vinylthiophene-terminated silicon quantum dots J Phys Chem C 2010 9 11693

[B39] Sommer A Cimpean C Kunz M Oelsner C Kupka HJ Kryschi C Ultrafast excitation energy transfer in vinylpyridine terminated silicon quantum dots J Phys Chem C 2011 9 22781

[B40] Atkins TM Thibert A Larsen DS Dey S Browning ND Kauzlarich SM Femtosecond ligand/core dynamics of microwave-assisted synthesized silicon quantum dots in aqueous solution J Am Chem Soc 2011 9 20664 22103236 10.1021/ja207344u PMC3377765

[B41] Rosso-Vasic M Cola LD Zuilhof H Efficient energy transfer between silicon nanoparticles and a Ru-polypyridine complex J Phys Chem C 2009 9 2235

[B42] Sudeep PK Emrick T Functional Si and CdSe quantum dots: synthesis, conjugate formation, and photoluminescence quenching by surface interactions ACS Nano 2009 9 4105 19908857 10.1021/nn901016u

[B43] Wang G Ji JW Xu XX Dual-emission of silicon quantum dots modified by 9-ethylanthracene J Mater Chem C 2014 9 1977

[B44] Dalton LK Demerac S Elmes BC Loder JW Swan JM Teitei T Synthesis of the tumour-inhibitory alkaloids, ellipticine, 9-methoxyellipticine, and related pyrido[4,3-b]carbazoles Aust J Chem 1967 9 2715

[B45] Thomas KRJ Lin JT Tao YT Ko CW Light-emitting carbazole derivatives: potential electroluminescent materials J Am Chem Soc 2001 9 9404 11562223 10.1021/ja010819s

[B46] Tsai MH Lin HW Su HC Ke TH Wu CC Fang FC Liao YL Wong KT Wu CI Highly efficient organic blue electrophosphorescent devices based on 3,6-bis(triphenylsilyl)carbazole as the host material Adv Mater 2006 9 1216

[B47] Tao YT Wang Q Yang CL Wang Q Zhang ZQ Zou TT Qin JG Ma DG A simple carbazole/oxadiazole hybrid molecule: an excellent bipolar host for green and red phosphorescent OLEDs Angew Chem Int Ed 2008 9 8104 10.1002/anie.200803396 18798180

[B48] Gale PA Synthetic indole, carbazole, biindole and indolocarbazole-based receptors: applications in anion complexation and sensing Chem Commun 2008 9 4525 10.1039/b809508f 18815678

[B49] Diaz-Garcia MA Wright D Casperson JD Smith B Glazer E Moerner WE Sukhomlinova LI Twieg RJ Photorefractive properties of poly(*N*-vinylcarbazole)-based composites for high-speed applications Chem Mater 1999 9 1784

[B50] Ikeda N Miyasaka T A solid-state dye-sensitized photovoltaic cell with a poly(*N*-vinyl-carbazole) hole transporter mediated by an alkali iodide Chem Commun 2005 9 1886 10.1039/b416461j 15795776

[B51] D'Angelo P Barra M Cassinese A Maglione MG Vacca P Minarini C Rubino A Electrical transport properties characterization of PVK (poly *N*-vinylcarbazole) for electroluminescent devices applications Solid State Electron 2007 9 123

[B52] Liu CY Holman ZC Kortshagen UR Hybrid solar cells from P3HT and silicon nanocrystals Nano Lett 2009 9 449 19113966 10.1021/nl8034338

[B53] Werwie M Xu XX Haase M Basché T Paulsen H Bio serves nano: biological light-harvesting complex as energy donor for semiconductor quantum dots Langmuir 2012 9 5810 22401299 10.1021/la204970a

[B54] Fujii T Kodaira K Kawauchi O Tanaka N Yamashita H Anpo M Photochromic behavior in the fluorescence spectra of 9-anthrol encapsulated in Si − Al glasses prepared by the sol–gel method J Phys Chem B 1997 9 10631

[B55] Xu XX Ji JW Wang G You XZ Exciton coupling of surface complexes on a nanocrystal surface Chem Phys Chem 2014 doi:10.1002/cphc.201402156 10.1002/cphc.201402156 24863364

[B56] Antwis L Gwilliam R Smith A Homewood K Jeynes C Characterization of a-FeSi_2_/c-Si heterojunctions for photovoltaic applications Semicond Sci Technol 2012 9 035016

[B57] Ritty JN Thomas KJ Jayasree VK Girijavallabhan CP Nampoori VPN Radhakrishnan P Study of solvent effect in laser emission from Coumarin 540 dye solution Appl Optics 2007 9 4786 10.1364/ao.46.004786 17609728

